# Robust detection of chromosomal interactions from small numbers of cells using low-input Capture-C

**DOI:** 10.1093/nar/gkx1194

**Published:** 2017-11-23

**Authors:** A. Marieke Oudelaar, James O.J. Davies, Damien J. Downes, Douglas R. Higgs, Jim R. Hughes

**Affiliations:** Medical Research Council (MRC) Molecular Haematology Unit, Weatherall Institute of Molecular Medicine, University of Oxford, Oxford OX3 9DS, UK

## Abstract

Chromosome conformation capture (3C) techniques are crucial to understanding tissue-specific regulation of gene expression, but current methods generally require large numbers of cells. This hampers the investigation of chromatin architecture in rare cell populations. We present a new low-input Capture-C approach that can generate high-quality 3C interaction profiles from 10 000–20 000 cells, depending on the resolution used for analysis. We also present a PCR-free, sequencing-free 3C technique based on NanoString technology called C-String. By comparing C-String and Capture-C interaction profiles we show that the latter are not skewed by PCR amplification. Furthermore, we demonstrate that chromatin interactions detected by Capture-C do not depend on the degree of cross-linking by performing experiments with varying formaldehyde concentrations.

## INTRODUCTION

The relationship between structural organization of the genome and regulation of gene expression is of considerable current interest. Chromosome conformation capture (3C) techniques play a key role in investigating how structural interactions between regulatory elements relate to gene activity (1). As these interactions are highly specific to cells of a particular tissue or developmental stage, it is crucial that 3C experiments are performed in well defined, purified cell populations.

A major limitation of all 3C techniques is the large numbers of cells required (2). To generate reproducible contact maps at high resolution using Hi-C, two to five million cells are required (3,4). The input for a 4C experiment is usually ten million cells (5), though UMI-4C can detect robust interaction profiles containing 5000–10 000 interactions using 1 μg of 3C library (6), which corresponds to ∼340 000 cells (assuming 50% loss during the 3C library preparation; Supplementary Figure S1). Next-Generation (NG) Capture-C is currently the most efficient 3C technique and can generate high-quality interaction profiles containing ∼19 000 interactions from 100 000 cells (7,8).

However, many primary tissues and rare cell populations are not available in these numbers and remain inaccessible for 3C analysis. We have therefore developed a new low-input Capture-C approach and show that we can generate high-quality interaction profiles from ∼20 000 cells at maximum resolution, and from ∼10 000 cells using windowing based analysis.

## MATERIALS AND METHODS

### Cells

The primary ter-119+ erythroid cells used in this study were obtained from the spleens of female C57BL/6 mice treated with phenylhydrazine (three doses of 40 mg/g body weight given 12 hours apart; mice were sacrificed after five days) as previously described (8). Mouse embryonic stem (ES) cells were derived from mice at embryonic day 14 and prepared as previously described (8).

### Low-input Capture-C experiments

The low-input Capture-C approach is described briefly below. A full protocol is available on Bio-protocol (9).

#### 3C library preparation

For the initial fixation step, ter-119+ erythroid cells were sorted into 100 μl RPMI + 10% FBS. Volumes were made up to 1 ml and fixed with 2% formaldehyde. Fixed and quenched cells were pelleted (500 g, 15 min) and washed with PBS. Following centrifugation, 5% supernatant was left behind to avoid disturbing the pellet. Centrifugation following cell lysis was omitted and lysed cells were snap frozen. Prior to digestion, cells were pelleted and lysis buffer was completely removed by careful pipetting. Chromatin was subsequently digested with the DpnII restriction enzyme in a 200 μl reaction, to which three doses of 150 U DpnII were added several hours apart and which was incubated overnight at 37°C. After heat-inactivation of the restriction enzyme, the ligation reaction was performed in the same tube, using 120 U T4 ligase in an overnight incubation at 16°C. After de-crosslinking, DNA was extracted using phenol-chloroform and transferred to a light phase lock tube for separation (15 000 g, 10 min). To maximize yield, DNA was precipitated overnight in 70% ethanol at –20°C, after which DNA was pelleted (21 000 g, 50 min, 4°C) and resuspended in PCR grade water.

The mass of DNA in the 3C libraries was quantified by qPCR relative to a standard curve generated from genomic DNA extracted from the same tissue (forward primer sequence: TTATCTTGCATTTGCCAACTCG; reverse primer sequence: TGGGTTTCCCTGATTCTGAAA).

To minimize losses, we did not assess digestion and ligation efficiency by using agarose gel electrophoresis of a digestion control and the 3C library. Instead, we determined digestion efficiency directly from the 3C library by comparing Ct values of qPCR analysis with primers spanning a ligation junction (forward primer sequence: GGAGAAAGAAGGCTGGTGTTAT; reverse primer sequence: TATCTGAGTTGGACAGCATTGG) and targeting a genomic control (forward primer sequence: TTATCTTGCATTTGCCAACTCG; reverse primer sequence: TGGGTTTCCCTGATTCTGAAA). All low-input libraries used in this study had a digestion efficiency >75%. Ligation efficiency of the 3C library was assessed using the Agilent Genomic ScreenTape system.

#### Low-Input Capture-C

3C libraries were sonicated to 200 bp fragments using a Covaris S220 Focused Ultrasonicator with the following settings: six cycles of 60 s; duty cycle, 10%; intensity, 5; cycles per burst, 200 (Supplementary Figure S2a). Illumina TruSeq adapters were subsequently added using the NEBNext Ultra DNA Library Prep kit according to the manufacturer's protocol. DNA clean up steps were performed with Ampure XP beads at a 1:1.8 ratio to minimize loss of material. The libraries were indexed and amplified in nine rounds of PCR amplification using the Herculase II PCR kit (Supplementary Figure S2b). 700 ng–1.5 μg of adaptor-ligated material was used for the oligonucleotide capture steps, which were performed as previously described, using oligonucleotides targeting the murine *Hba-a1, Hba-a2, Hbb-b1, Hbb-b2* and *Slc25a37* promoters (8).

#### Tag-Capture-C

3C libraries were tagmented using reagents from the Nextera DNA Library Prep kit. To generate fragments within our desired size range (Supplementary Figure S2c), tagmentation conditions were optimized: a maximum input of 50 ng 3C library was used per reaction and reaction time was extended to overnight incubation at 55°C. The tagmentation reactions were cleaned up using the Zymo DNA Clean & Concentrator system. When input was >50 ng, several tagmentation reactions were performed in parallel and pooled after the clean up. The libraries were amplified in nine rounds of PCR using the KAPA Hi-Fi PCR kit (Supplementary Figure S2d), as this generated a higher yield per cycle compared to the PCR strategy used in the Nextera DNA Library Prep kit. 1.5 μg of tagmented material was used for the oligonucleotide capture steps targeting the murine *Hba-a1, Hba-a2, Hbb-b1, Hbb-b2* and *Slc25a37* promoters. Tag-Capture-C libraries contain sequencing adapters that are not compatible with commercially available blocking oligonucleotides. These oligonucleotides are used to prevent concatemerization through annealing of the adaptor sequences (‘daisy chaining’), which markedly reduces hybridization efficiency. We therefore designed custom blocking oligonucleotides (xGen Custom Blocking Oligos, IDT). The rest of the oligonucleotide capture procedure was performed as previously described (8).

#### Sequencing

All Capture-C libraries were sequenced using Illumina platforms. We used at least 500k 150 bp paired-end reads per viewpoint, per sample in the multiplexed libraries to produce sufficient sequencing depth.

#### Data analysis and duplicate filtering

The approach for data analysis was based on the NG-Capture-C analysis pipeline (8). Removal of PCR duplicates in this analysis strategy depends on using the random unique ends of the reads that result from the sonication or tagmentation step as two independent unique molecular identifiers (UMIs). Reads with exactly the same mapped start and end coordinates were excluded from analysis. However, occasional sequencing and/or mapping errors can cause ‘wobbly’ ends and make PCR duplicates seem unique. In low-input libraries, in which complexity is limited, this can cause spurious peaks in interaction profiles. We therefore created a more stringent duplicate filter, in which all reads with one or two differences in the UMIs were also excluded from analysis. While this approach is very conservative and will also remove genuine unique fragments, therefore giving an underrepresentation of the number of unique interactions detected by the low-input Capture-C protocols, it ensures detection of robust interactions only.

For all Capture-C experiments a mixture of capture oligonucleotides targeting the murine *Hba-a1, Hba-a2, Hbb-b1, Hbb-b2* and *Slc25a37* promoters was used. Because interactions with the duplicated α- and β-globin promoters cannot be distinguished bioinformatically, the figures show composite interaction profiles in which the data from the duplicated promoter viewpoints are pooled.

### C-String

#### Design

NanoString technology was adapted to quantify interactions in 3C libraries by designing capture oligonucleotides to target the ends of the restriction fragments containing the α-globin promoters, which were used as the viewpoint for the experiments. The reporter oligonucleotides were designed to target 120 restriction fragments across the α-globin locus, including fragments that contain regulatory elements and multiple control regions. This design allowed for direct quantification of the ligation junctions present in the 3C library between the α-globin promoters and the restriction fragments to which reporter probes had been designed. All the sequences were designed using the top strand of the left end and the bottom strand of the right end of the fragments.

#### Experimental procedure

3C libraries were prepared from aliquots of 10 million murine primary ter-119+ erythroid and ES cells in biological duplicates as described above. To assess the accuracy of the quantification by the NanoString system, experiments were performed with 5 μg, 2 μg and 500 ng 3C library input. The 3C libraries were sonicated to 200 bp and purified using Ampure XP beads as described above. Vacuum centrifugation at 50°C was used to concentrate the libraries to a volume of 5 μl. Libraries were subsequently denatured by heating to 95°C for 10 min, and incubated with the NanoString code set at 65°C for 18 h, to allow the capture and reporter probes to anneal to the 3C template. After the hybridization reaction, the material was loaded onto the NanoString cartridge using the nCounter Prep Station. The cartridge was analyzed by the nCounter Digital Analyser using the settings for maximum resolution.

### Alteration of fixation conditions

Primary ter-119+ erythroid cells were obtained from spleens of phenylhydrazine treated mice as described above. Aliquots of 10 million cells in 10 ml media were fixed with 5%, 4%, 3%, 2%, 1% or 0.5% formaldehyde for 10 minutes at room temperature. The reaction was quenched with 1.5 ml 1 M glycine. These samples were washed with 10 ml PBS, along with an aliquot of unfixed material. Following centrifugation, pellets were resuspended in 5 ml lysis buffer (8), incubated on ice for 20 min and snap frozen. 3C library preparation was performed as previously described (8), except that the sample of unfixed material was Dounce homogenized in the volume of water required for the digestion reaction and the subsequent centrifugation step was omitted. Digestion efficiencies in the generated 3C libraries were calculated by qPCR analysis as described above. Library preparation, oligonucleotide capture and sequencing were performed as previously described, using a mixture of capture oligonucleotides targeting the murine *Hba-a1, Hba-a2, Hbb-b1, Hbb-b2* and *Slc25a37* promoters (8).

## RESULTS

### Low-input Capture-C approaches can generate high-quality interaction profiles from small numbers of cells

The Capture-C protocol contains two critical stages in which significant data loss may occur: preparation of the 3C library—a common step in all 3C experiments—and processing of this material into a sequencing library, that is subsequently amplified, captured and sequenced. The efficiency of these two stages determines the minimal number of cells required. We optimized both stages independently, which allowed us to carefully compare different approaches and quantify gains and losses accurately at every step. We subsequently combined the optimized stages into a single protocol, and performed the entire procedure directly on low-input samples to demonstrate our new approach.

Optimization of the 3C library preparation included adaptation of the protocol to a single-tube procedure to prevent DNA losses during digestion and ligation of chromatin and the use of Phase Lock Gel technology for DNA recovery. Using this protocol, we generated 3C libraries from 100 000, 50 000, 20 000 and 10 000 cells, and were able to recover ∼50% of the DNA mass, irrespective of the cell number used (Supplementary Figure S1).

For the subsequent interrogation of the 3C libraries, we developed two new Capture-C based approaches: Low-Input (LI) Capture-C and Tag-Capture-C. These approaches use different sequencing library preparation protocols, which are more suitable for the reduced input. We optimized both protocols using triplicate aliquots of 200, 100 or 50 ng 3C library, corresponding to ∼68 000, ∼34 000 and ∼17 000 cells, respectively (Supplementary Figure S1). We tested the protocols by analyzing the well-characterized α-globin (*Hba-a1* & *Hba-a2*), β-globin (*Hbb-b1* & *Hbb-b2*) and Mitoferrin-1 (*Slc25a37*) loci in primary murine erythroid cells.

**Figure 1.**
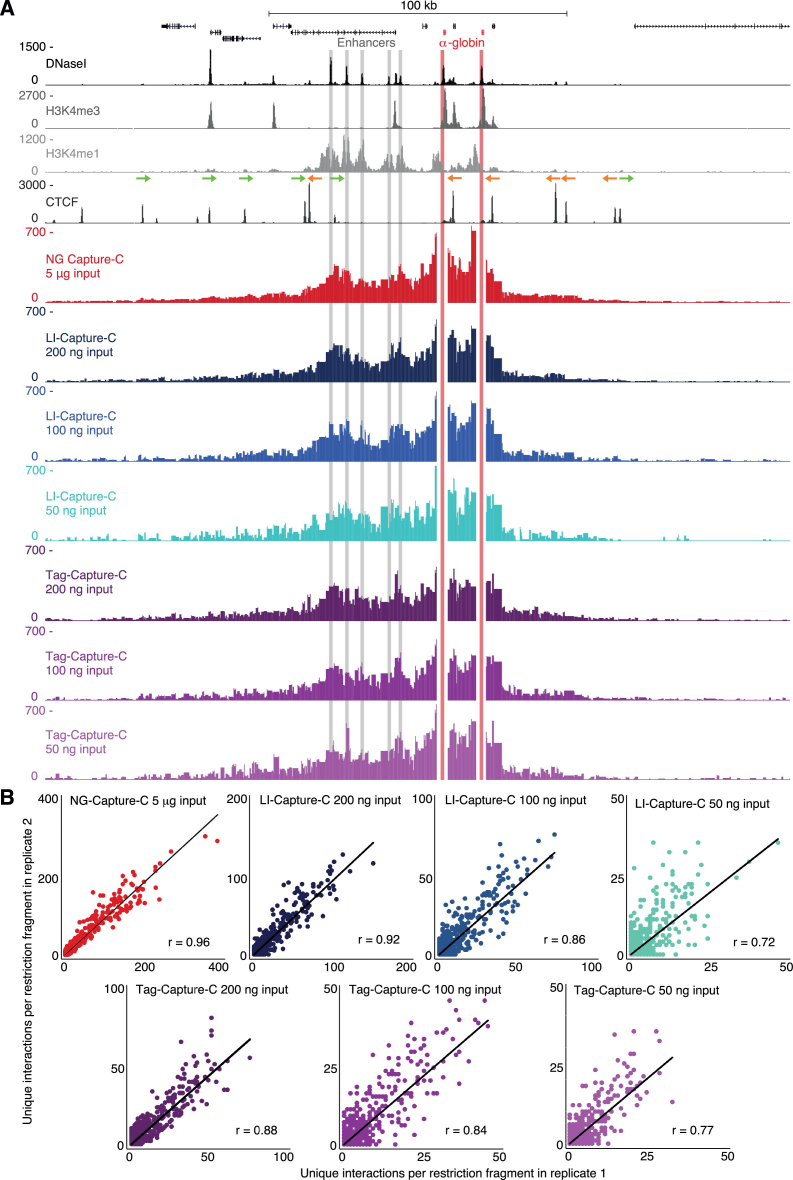

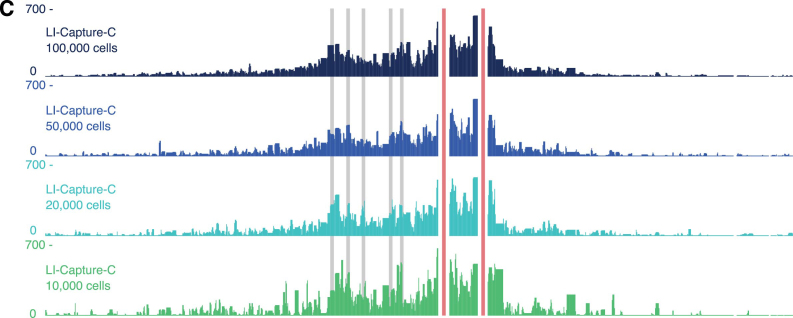
LI-Capture-C and Tag-Capture-C can generate reproducible, high-quality interaction profiles from small numbers of cells. (**A**) Comparison of interaction profiles from the viewpoint of the α-globin promoters (highlighted in red) generated from decreasing amounts of 3C libraries (prepared from primary erythroid cells) with NG Capture-C (red), LI-Capture-C (blue) and Tag-Capture-C (purple). DNaseI hypersensitivity, H3K4me3, H3K4me1 and CTCF occupancy are shown at the top, with the orientation of the CTCF-binding sites (16) indicated by arrows. Capture-C profiles show the mean number of unique interactions per restriction fragment from three replicates, normalized for a total of 100 000 interactions genome-wide. The α-globin enhancers are highlighted in gray. (**B**) Correlation of chromosomal interactions detected by NG Capture-C, LI-Capture-C and Tag-Capture-C in individual replicates. The *r*-values represent Pearson correlation coefficients. (**C**) LI Capture-C interaction profiles from the viewpoint of the α-globin promoters (highlighted in red) generated from decreasing numbers of primary erythroid cells. Profiles represent the mean number of unique interactions per restriction fragment from three replicates, normalized for a total of 100 000 interactions genome-wide. The α-globin enhancers are highlighted in gray.

The LI-Capture-C protocol uses a conventional sequencing library preparation strategy based on the NEBNext Ultra DNA Library Prep kit, which involves sonication and enzymatic end-repair, dA-tailing and adaptor ligation (Supplementary Figure S2). When using 200 ng and 100 ng 3C libraries, the LI-Capture-C interaction profiles were highly reproducible at maximum resolution (individual restriction fragments), with Pearson correlation coefficients (*r*-values) for the different viewpoints between 0.86–0.92 and 0.76–0.86, respectively (Figure 1A and B; Supplementary Figures S3–S6). Although the interaction profiles generated from 50 ng samples were noisier and less reproducible (*r*-values between 0.52 and 0.72), the profiles were still highly informative and interpretable, especially when the data were represented in windowed interaction profiles (as routinely performed in 4C analysis (5); Supplementary Figure S7). An overview of the number of interactions detected by LI-Capture-C is shown in Table 1. Because incomplete PCR duplicate filtering can cause skewing in profiles generated from low cell numbers, the interaction numbers shown here represent the unique interactions after extremely stringent duplicate filtering (Materials and Methods).

**Table 1.** Unique interactions detected per viewpoint using NG Capture-C, LI-Capture-C and Tag-Capture-C

	*Hba-1*	*Hba-2*	*Hbb-1*	*Hbb-2*	*Slc25a37*
NG Capture-C 5 μg input	169 744	166 114	122 794	157 370	119 985
LI-Capture-C 200 ng input	14 012	14 131	12 548	11 730	9035
LI-Capture-C 100 ng input	6824	6749	5713	5350	4251
LI-Capture-C 50 ng input	2929	2903	2549	2280	1978
Tag-Capture-C 200 ng input	7414	7436	8059	8354	6968
Tag-Capture-C 100 ng input	4769	4720	5011	5443	4417
Tag-Capture-C 50 ng input	2645	2633	2908	3170	2606

Table shows the average of three biological replicates of erythroid cells.

Compared to the multi-step conventional library preparation strategy, the one-step transposase-catalyzed adaptor insertion (‘tagmentation’) has been presented as a more efficient method of sequencing library preparation (10). To investigate if this approach could further reduce 3C input requirements, we developed a tagmentation based Capture-C protocol, which we termed Tag-Capture-C. Comparison to the LI-Capture-C approach (Figure 1A–B; Supplementary Figures S3–S6; Table 1) showed that interaction profiles generated with Tag-Capture-C from 100–200 ng 3C libraries were less reproducible (*r*-values between 0.84–0.88 and 0.82–0.84, respectively) and contained fewer unique interactions. However, when only 50 ng of 3C library was used, Tag-Capture-C outperformed LI-Capture-C, detecting more unique interactions for most viewpoints, with higher reproducibility between samples (*r*-values between 0.71 and 0.77).

The lower performance of Tag-Capture-C in the samples with 100–200 ng input is likely related to the fact that tagmentation events, unlike sonication breakpoints, are not randomly distributed over the genome (Supplementary Figure S8a). The preference for C and G bases (11,12) makes it more likely that independent tagmentation events in different cells take place at exactly the same genomic locations in and around targeted restriction sites, which results in fragments with identical genomic coordinates that are indistinguishable from PCR duplicates and removed from analysis. The probability of independent tagmentation events occurring at identical genomic locations decreases when fewer cells are sampled, which could explain why the efficiency of Tag-Capture-C improves when the amount of input DNA is reduced. The differences in performance between LI-Capture-C and Tag-Capture-C are unlikely to be related to the differences in fragment size distribution of the libraries, as the number of detected restriction fragments per read is very similar in LI-Capture-C and Tag-Capture-C libraries (Supplementary Figure S8b).

Importantly, lowering input does not influence the throughput of Capture-C, as the quality of interaction profiles reflects the complexity of the Capture-C libraries and does not depend on the number of genomic regions sampled (8). The low-input Capture-C approaches therefore allow for multiplexing hundreds of viewpoints at high resolution (restriction enzyme with 4-base-pair recognition sequence) or thousands at lower resolution (restriction enzyme with 6-base-pair recognition sequence) and for analysis of dozens of samples in a single experiment. Here, we analyzed the α-globin, β-globin and Mitoferrin-1 loci simultaneously, which all performed similarly for both low-input protocols (Supplementary Figures S3–S6).

To demonstrate the effectiveness of our protocols, we performed the entire LI Capture-C procedure on 10 000, 20 000, 50 000 and 100 000 cells. These experiments confirm that high-quality interactions profiles can be generated from very small numbers of cells (Figure 1C; Supplementary Figure S9).

### Quantitative C-String analysis shows that interaction frequencies defined by Capture-C are accurate and not skewed by PCR amplification

Direct comparison of LI-Capture-C and Tag-Capture-C shows very similar interaction profiles (*r* = 0.88 for the α-globin locus when using 200 ng input; Supplementary Figure S4), suggesting that the sequencing library preparation strategy does not introduce a systematic skew in the profiles. However, both methods involve extensive PCR amplification that could bias the detected interactions. This is a major concern for all 3C based methods, especially when library complexity is limited due to reduced input, as in our current application.

**Figure 2.**
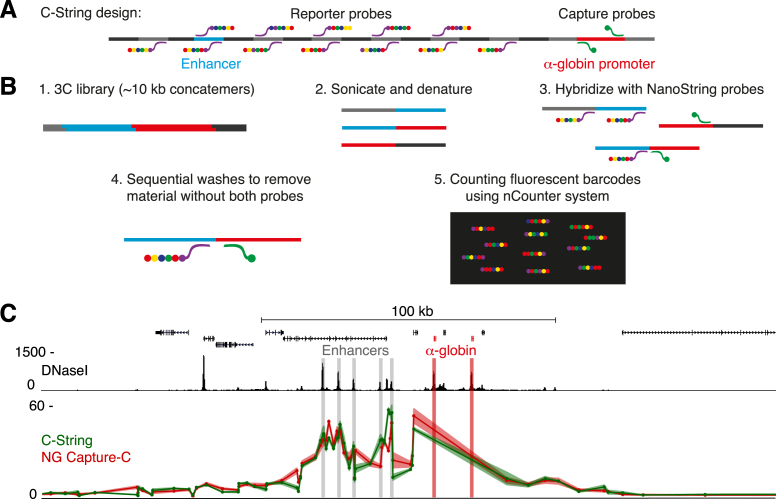
Direct quantification of 3C interactions by C-String demonstrates that Capture-C interaction profiles are not skewed by PCR amplification. (**A**) Overview of C-String design. NanoString capture probes are targeted to the restriction fragment containing the α-globin promoters, and reporter probes are designed to target 120 restriction fragments across the α-globin locus, including fragments containing regulatory elements and multiple control regions. (**B**) Overview of C-String experimental procedure. The large concatemers in the 3C library (1) are sonicated to ∼200 bp fragments and denatured to produce single stranded DNA (2), to which the NanoString probes are hybridized (3). After sequential washes to remove material without both the capture and reporter probes (4), the barcodes on the probes are counted (5), allowing for precise quantification of interactions between the reporters and the α-globin promoters. (**C**) Interaction frequencies between the α-globin promoters (highlighted in red) and the restriction fragments analyzed by C-String (5 μg input; green) in erythroid cells, compared to the corresponding fragments analyzed by NG Capture-C (red). The data points in the profiles represent the mean number of interactions from two and three replicates, respectively, normalized for a total of 1000 interactions, and are connected by a line, with the standard error indicated by the line shadow.

To assess the potential PCR related bias, we developed a PCR-free, sequencing-free 3C technique called C-String, which uses NanoString nCounter technology (13) to directly detect interactions in 3C libraries. As NanoString technology uses hybridization of barcoded probes and single molecule imaging to count nucleic acid molecules, and does not involve any amplification steps, C-String allows for direct quantification of ligation junctions in 3C libraries. We used a design that allowed us to accurately quantify the interactions between the α-globin promoters and 120 restriction fragments in the surrounding locus, including fragments containing the regulatory elements and multiple control regions (Figure 2A and B). To assess the precision of C-String, we compared interaction counts using 5, 2 and 0.5 μg 3C library input, in both erythroid and ES cells. The resulting counts were highly reproducible between technical replicates, and there was a linear relation between quantity of input library and detected NanoString counts, showing that C-String is highly quantitative (Supplementary Figure S10). C-String has the additional advantages that it allows for multiplexing up to twelve samples and that data analysis is much more straightforward than in sequencing based 3C experiments.

Comparison of the C-String interaction profile in erythroid cells to the normalized number of interactions detected by NG Capture-C for the corresponding restriction fragments shows a high degree of similarity and correlation (*r* = 0.94; Figure 2C). Differential interaction profiles, in which the interactions in the inactive ES cells are subtracted from interactions in erythroid cells to highlight relevant tissue-specific interactions, are even more similar (Supplementary Figure S11). The very strong coherence between profiles generated using Capture-C and the PCR-free C-String approach shows that analysis by Capture-C introduces negligible skew in the quantification of detected interactions. This is likely due to the use of highly optimized PCR conditions in Capture-C, in which uniformly sized fragments are amplified with the Illumina P5 and P7 primers, and the ability to strictly control for PCR duplication based on the two random sonicated fragment ends that act as UMIs.

### Capture-C interaction profiles are not skewed by the degree of formaldehyde fixation

Another general concern in the 3C field is the potential bias resulting from cross-linking, which could cause a skew in interaction profiles generated from samples with limited material in which the degree of cross-linking is likely to vary (14). The most widely used 3C methods use formaldehyde fixation (Hi-C: 1% formaldehyde; 4C and Capture-C: 2% formaldehyde) to cross-link chromatin (3,5,7). Though it has been shown by Hi-C (4) and intrinsic 3C (i3C) (15) that formaldehyde cross-linking does not affect the pattern of chromosomal interactions detected at larger scale, the effects of varying levels of crosslinking on interaction profiles displayed at maximum resolution are still unclear.

**Figure 3.**
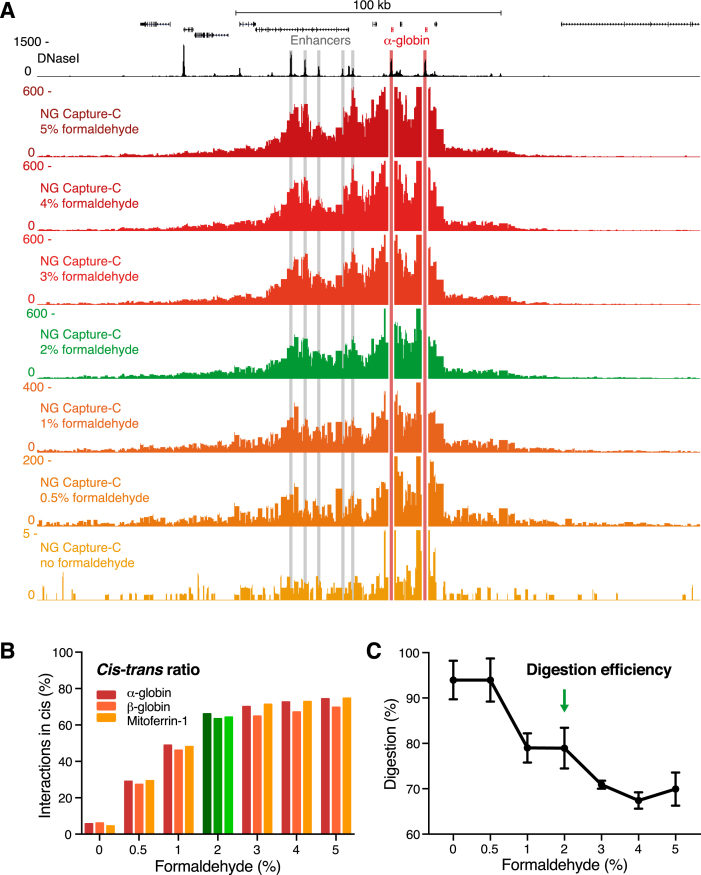
Capture-C in samples fixed with varying formaldehyde concentrations shows that detection of chromatin interactions does not depend on the degree of crosslinking. (**A**) Comparison of NG Capture-C interaction profiles generated from the viewpoint of the α-globin promoters in primary erythroid cells that have been fixed with different concentrations of formaldehyde. Profiles show the mean number of unique interactions per restriction fragment from technical duplicates, normalized for a total of 100 000 interactions genome-wide, with the scales adjusted for the different conditions. (**B**) Percentage of unique interactions detected in *cis* in samples fixed with varying formaldehyde concentrations. Percentages represent an average of two technical replicates. (**C**) Digestion efficiencies in 3C libraries generated from samples fixed with varying formaldehyde concentrations. Graph represents the mean and standard error of two technical replicates.

To assess if Capture-C interaction profiles depend on the degree of cross-linking, we performed experiments in which we fixed samples of erythroid cells using formaldehyde concentrations ranging between 0% and 5%. The detected interaction patterns in these samples were very similar (Figure 3A, Supplementary Figures S12 and S13). However, reduction of formaldehyde <2% decreased the number of informative interactions detected (Figure 3A; indicated by adjusted scaling). This decrease in specific signal was associated with an increase in *trans* interactions genome-wide (Figure 3B). Fixing with more than 2% formaldehyde only marginally improved the *cis-trans* ratios. Importantly, cells fixed to such a high degree were more difficult to digest (Figure 3C). Even though this is not directly reflected in interaction profiles that are generated from millions of cells and normalized for the total number of interactions detected, it reduces library complexity, which is critical when lower numbers of cells are used.

Taken together, our analyses show that the pattern of Capture-C interaction profiles is independent of the degree of fixation and therefore not an artifact of the fixation process. However, the efficiency with which interactions are captured and the degree of noise depend on adequate levels of fixation. We recommend fixing with 2% formaldehyde, and careful adjustment for tissues that respond differently to formaldehyde to optimize *cis-trans* ratios and digestion efficiencies.

## DISCUSSION

In summary, these results show that interactions detected by Capture-C are not skewed by PCR amplification or cross-linking conditions. Moreover, we demonstrate that the optimized LI-Capture-C and Tag-Capture-C protocols are able to generate high-quality interaction profiles from 10 000 to 20 000 cells, depending on the resolution used for analysis. These new approaches allow for the generation of highly multiplexed 3C interaction profiles at high resolution from rare tissues and cell types which are currently intractable.

## AVAILABILITY

Scripts for analysis of low-input Capture-C experiments are available in the GitHub repository (https://github.com/oudelaar/CaptureC/). Analyzed LI-Capture-C and Tag-Capture-C data for the α-globin locus are available in a UCSC Genome Browser Track Hub (http://sara.molbiol.ox.ac.uk/public/hugheslab/LowInputCaptureCHub/hub.txt). Sequencing data have been deposited in the NCBI Gene Expression Omnibus (GEO; http://www.ncbi.nlm.nih.gov/geo/) under accession number GSE99257.

## Supplementary Material

Supplementary Data
