# Supplementary material for: Robust detection of chromosomal interactions from small numbers of cells using low-input Capture-C
Source: Nucleic Acids Res. 2017 Nov 23;45(22):e184. doi: 10.1093/nar/gkx1194 (PMC5728395; doi:10.1093/nar/gkx1194)
Supplement: Supplementary Data [file gkx1194_supp.pdf]

Supplementary Figure 1

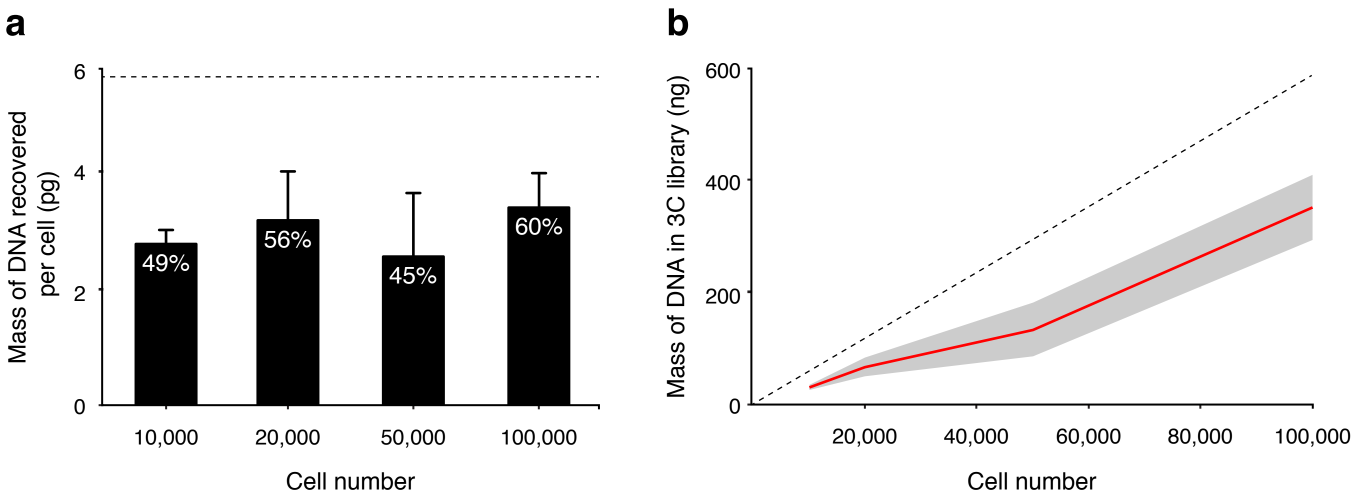

Supplementary Figure 1. 3C library preparation efficiency.

(a) 3C library preparation efficiency expressed as mass of DNA recovered per cell. The bars represent the average and standard error of five technical replicates of 10,000–100,000 primary erythroid cells. The dashed line indicates the theoretical maximum of 5.86 pg, which is the weight of the diploid mouse genome. The percentages indicate the mass recovered relative to this maximum.

(b) Relationship between the number of cells used as input for the 3C library preparation protocol and the mass of DNA in the generated 3C library. The red line shows the average 3C library mass generated from five technical replicates of 10,000, 20,000, 50,000 or 100,000 primary erythroid cells, with the standard error indicated by the grey line shadow, and the theoretical maximum indicated by the dashed line.

Supplementary Figure 2

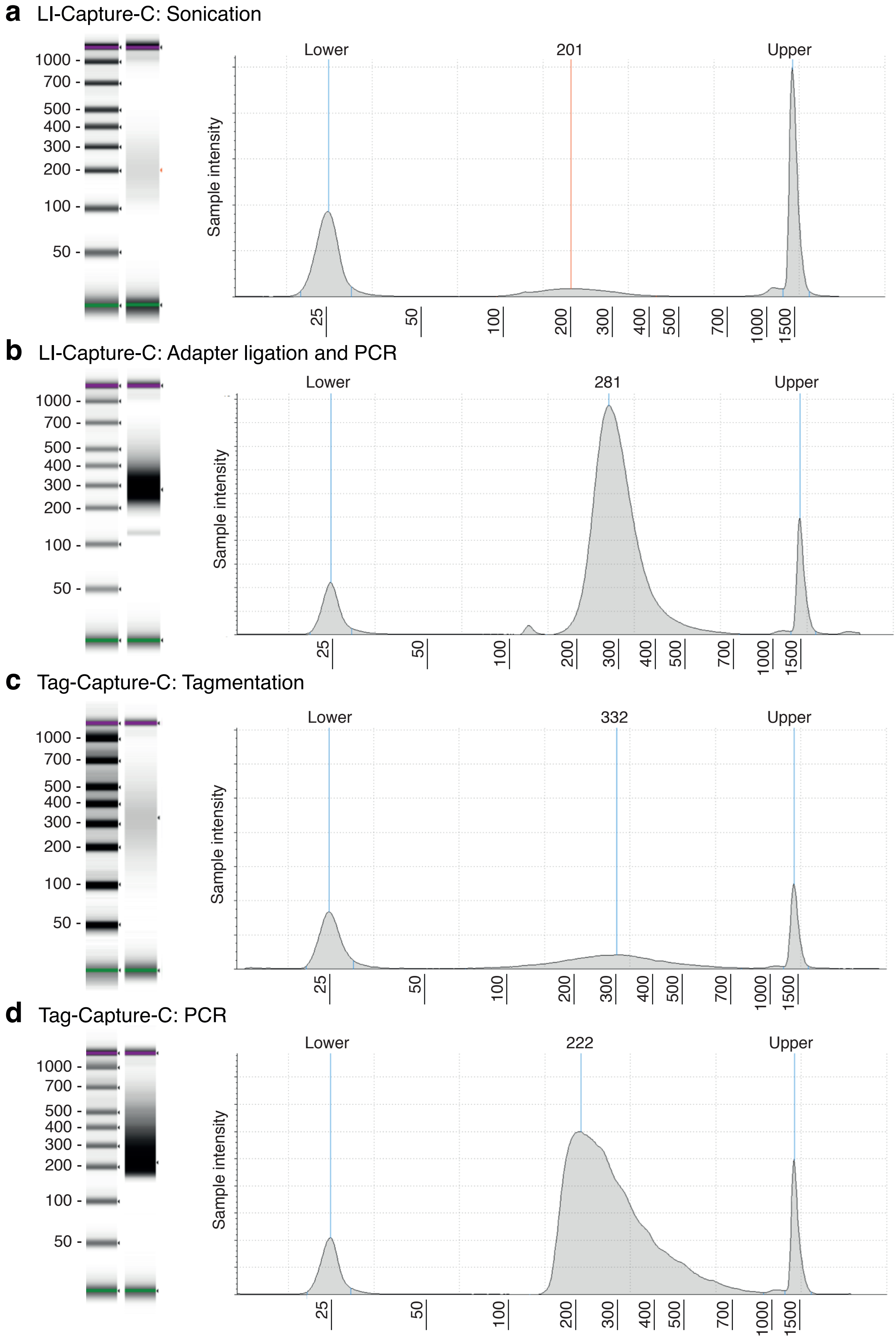

Supplementary Figure 2. Sequencing library preparation in LI-Capture-C and Tag-Capture-C. Assessment of the 3C library material using the Agilent ScreenTape system after **(a)** sonication and **(b)** adapter ligation and amplification, as part of the LI-Capture-C protocol; and **(c)** tagmentation and **(d)** amplification, as part of the Tag-Capture-C protocol.

Supplementary Figure 3

a

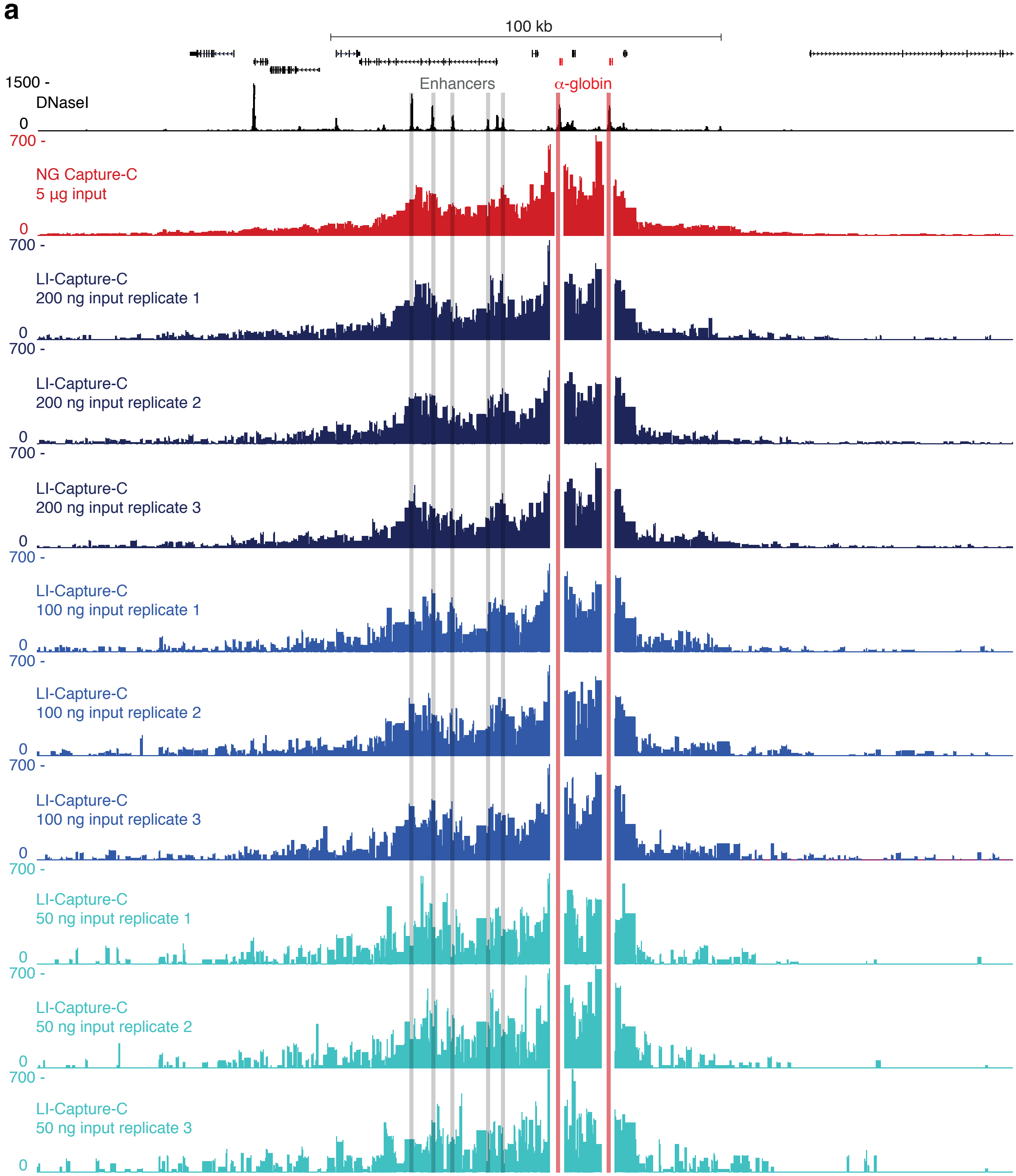

(Legend on next page.)

Supplementary Figure 3

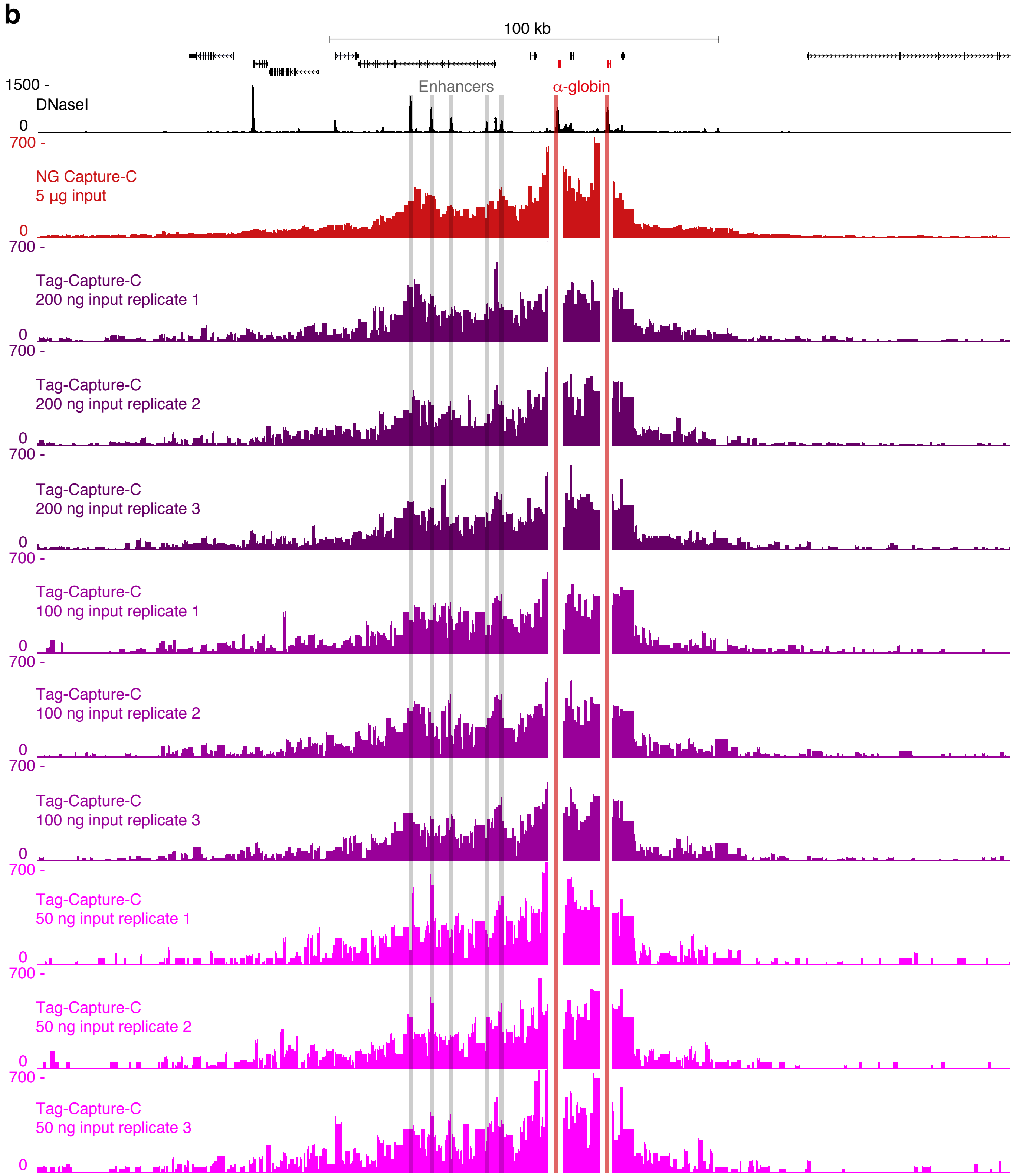

Supplementary Figure 3. Reproducibility of LI-Capture-C and Tag-Capture-C interaction profiles in biological replicates.
Interaction profiles from the viewpoint of the α-globin promoters (highlighted in red) from individual replicates, generated from decreasing amounts of 3C libraries (prepared from primary erythroid cells) with **(a)** LI-Capture-C and **(b)** Tag-Capture-C. Profiles show the number of unique interactions per restriction fragment, normalized for a total of 100,000 interactions genome-wide. The α-globin enhancers are highlighted in grey.

Supplementary Figure 4

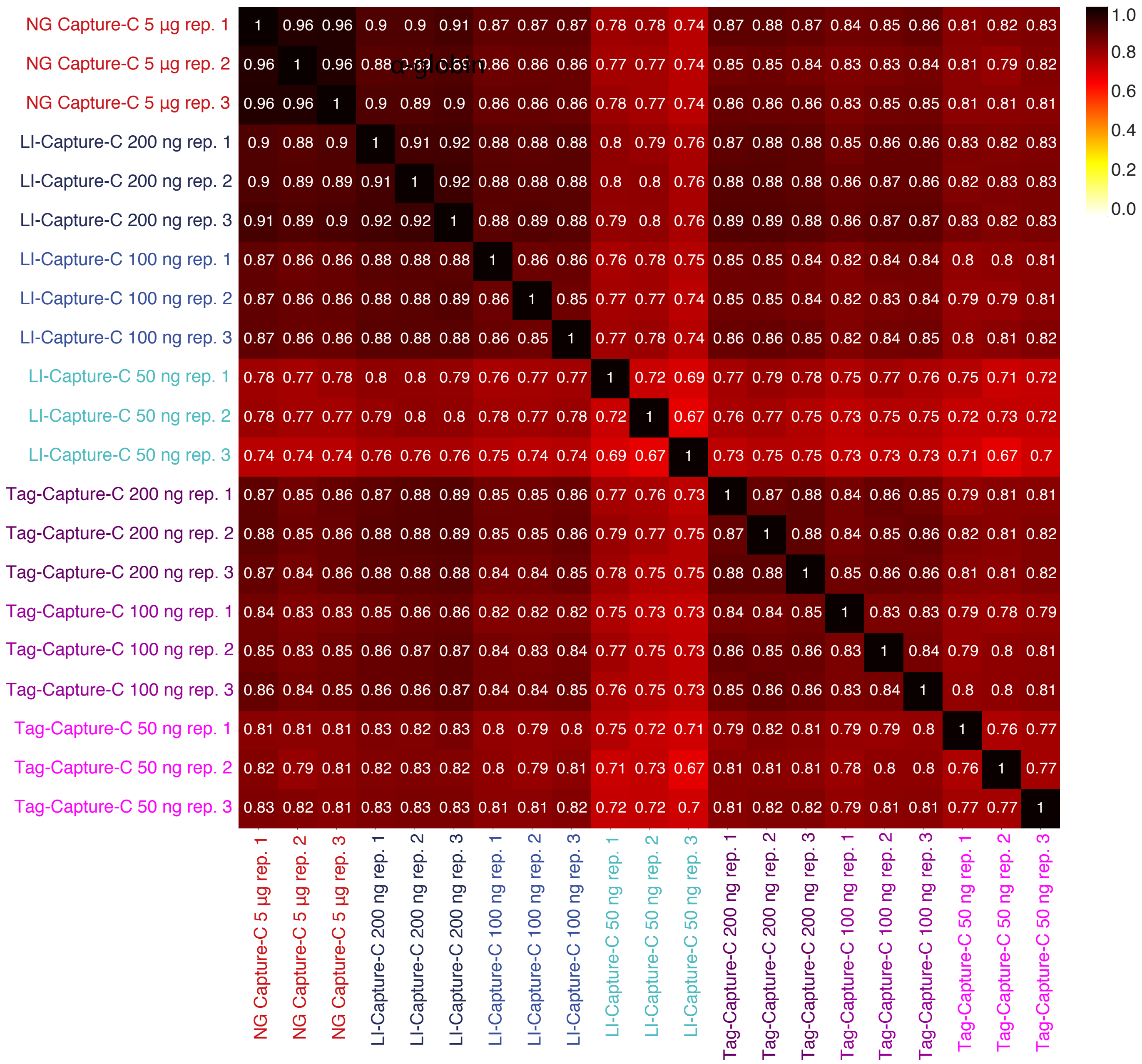

Supplementary Figure 4. Correlation between interaction profiles of the α-globin locus from individual replicates generated by different Capture-C protocols.
Heatmap showing the correlation between the α-globin interaction profiles generated by NG Capture-C, LI-Capture-C and Tag-Capture-C. Numbers represent Pearson correlations coefficients.

Supplementary Figure 5

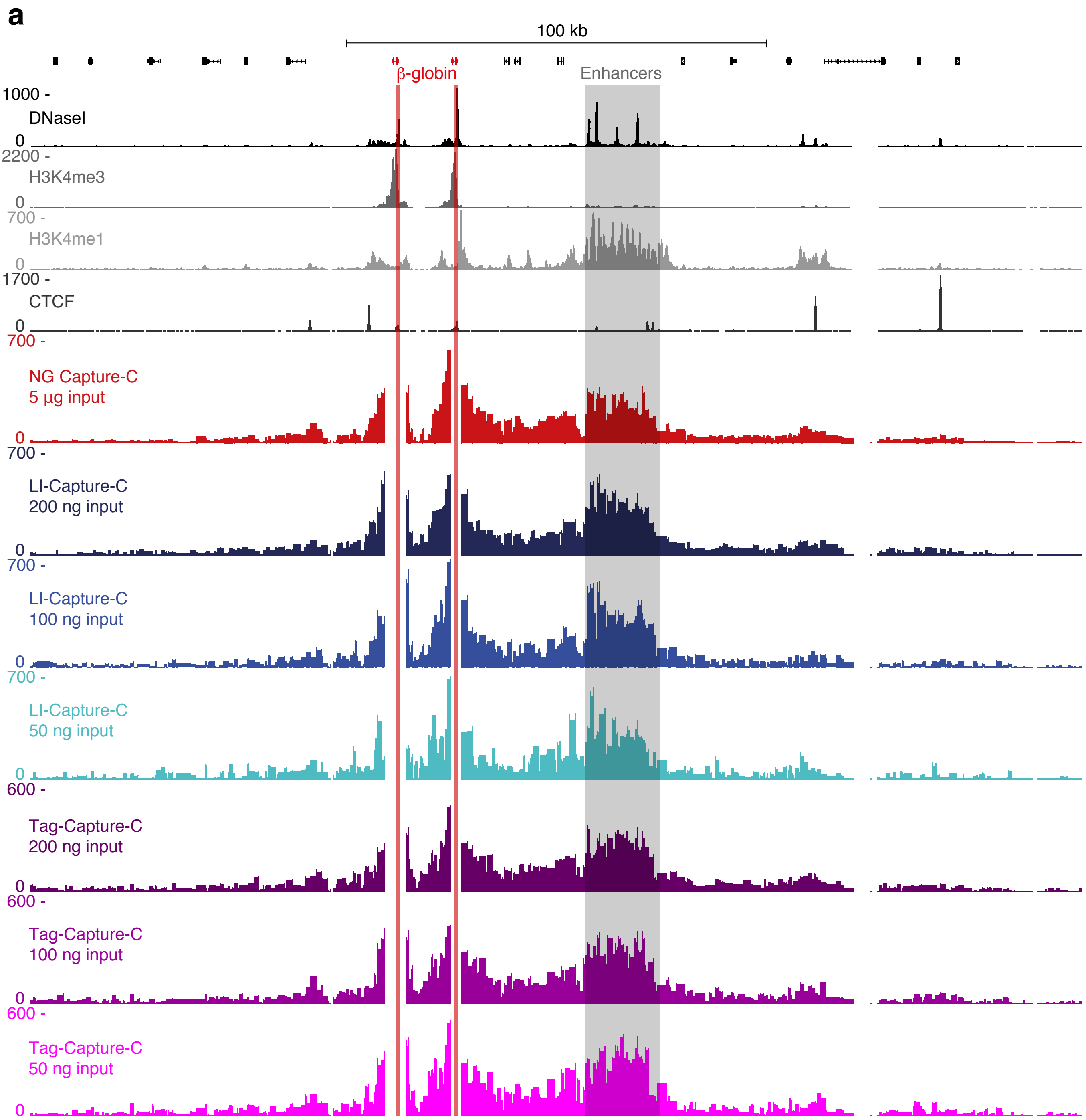

(Legend on next page.)

Supplementary Figure 5

b

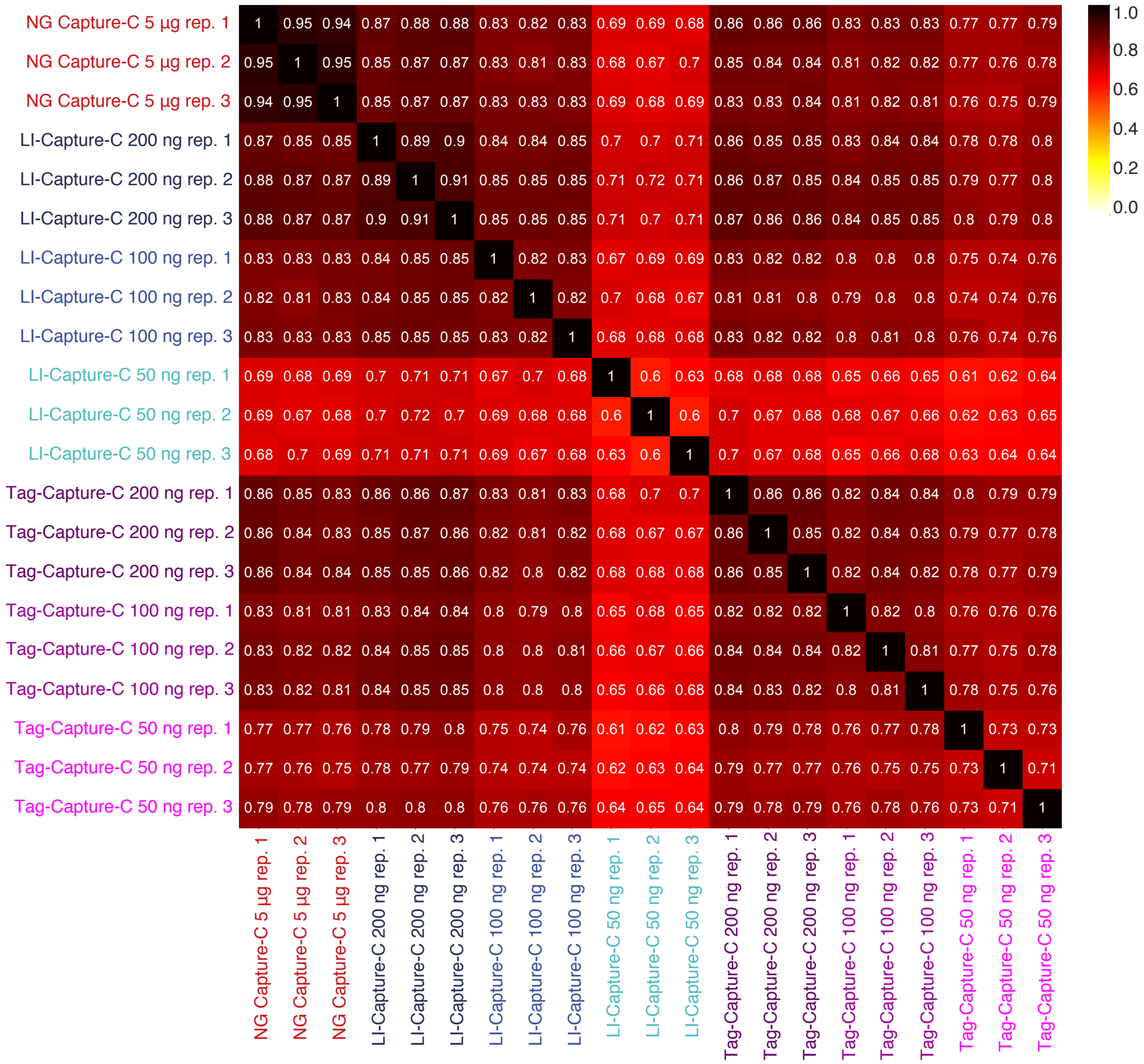

Supplementary Figure 5. Comparison of NG Capture-C, LI-Capture-C and Tag-Capture-C interaction profiles of the β-globin locus.

(a) Comparison of interaction profiles from the viewpoint of the β-globin promoters (highlighted in red) generated from decreasing amounts of 3C libraries (prepared from primary erythroid cells) with NG Capture-C (red), LI-Capture-C (blue) and Tag-Capture-C (purple). Profiles show the mean number of unique interactions per restriction fragment from three replicates, normalized for a total of 100,000 interactions genome-wide. The β-globin enhancers are highlighted in grey.

(b) Heatmap showing the correlation between the β-globin interaction profiles generated by NG Capture-C, LI-Capture-C and Tag-Capture-C. Numbers represent Pearson correlations coefficients.

Supplementary Figure 6

a

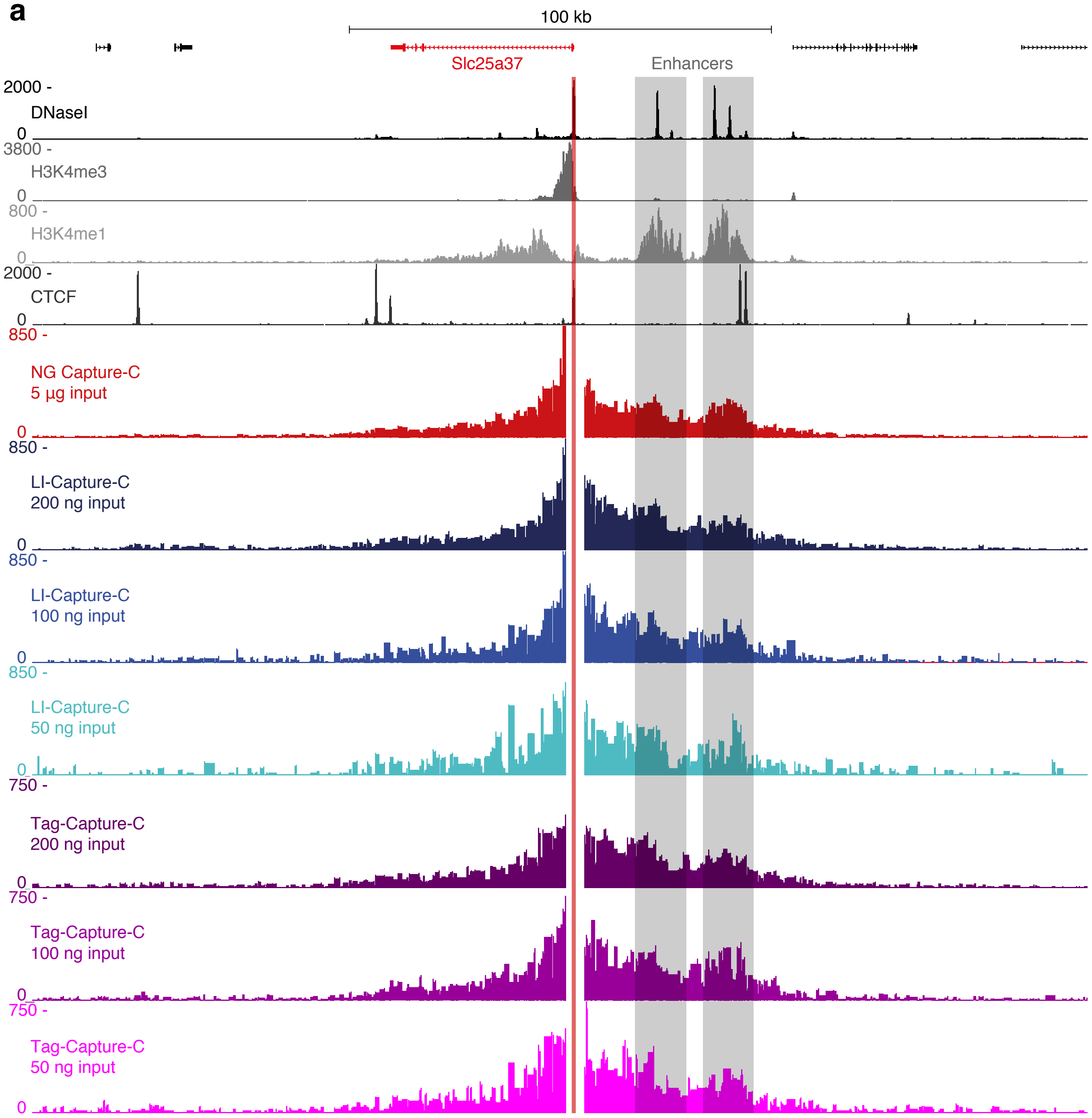

(Legend on next page.)

Supplementary Figure 6

b

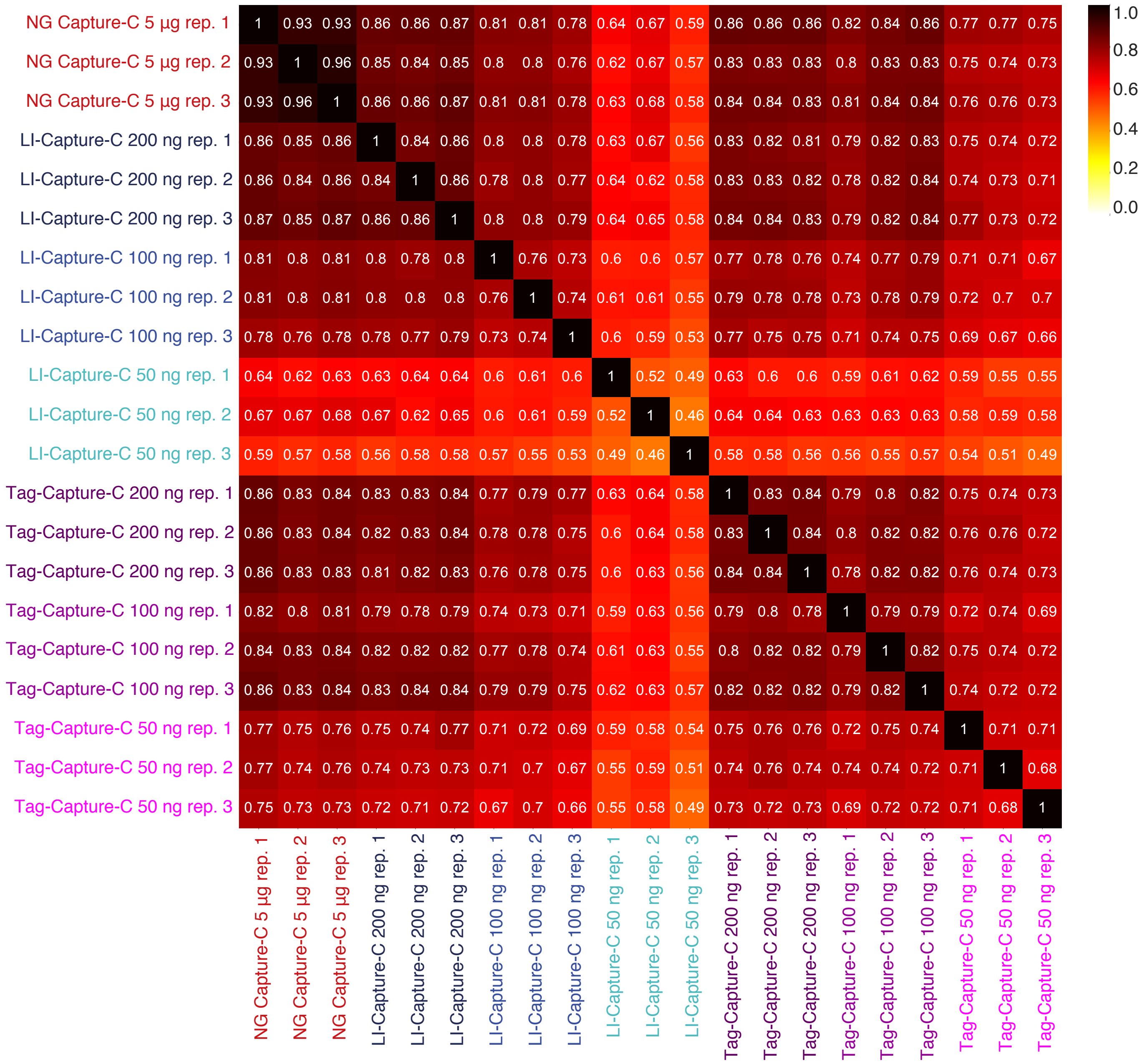

Supplementary Figure 6. Comparison of NG Capture-C, LI-Capture-C and Tag-Capture-C interaction profiles of the Mitoferrin-1 locus.

(a) Comparison of interaction profiles from the viewpoint of the Mitoferrin-1 promoter (highlighted in red) generated from decreasing amounts of 3C libraries (prepared from primary erythroid cells) with NG Capture-C (red), LI-Capture-C (blue) and Tag-Capture-C (purple). Profiles show the mean number of unique interactions per restriction fragment from three replicates, normalized for a total of 100,000 interactions genome-wide. The Mitoferrin-1 enhancers are highlighted in grey.

(b) Heatmap showing the correlation between the Mitoferrin-1 interaction profiles generated by NG Capture-C, LI-Capture-C and Tag-Capture-C. Numbers represent Pearson correlations coefficients.

Supplementary Figure 7

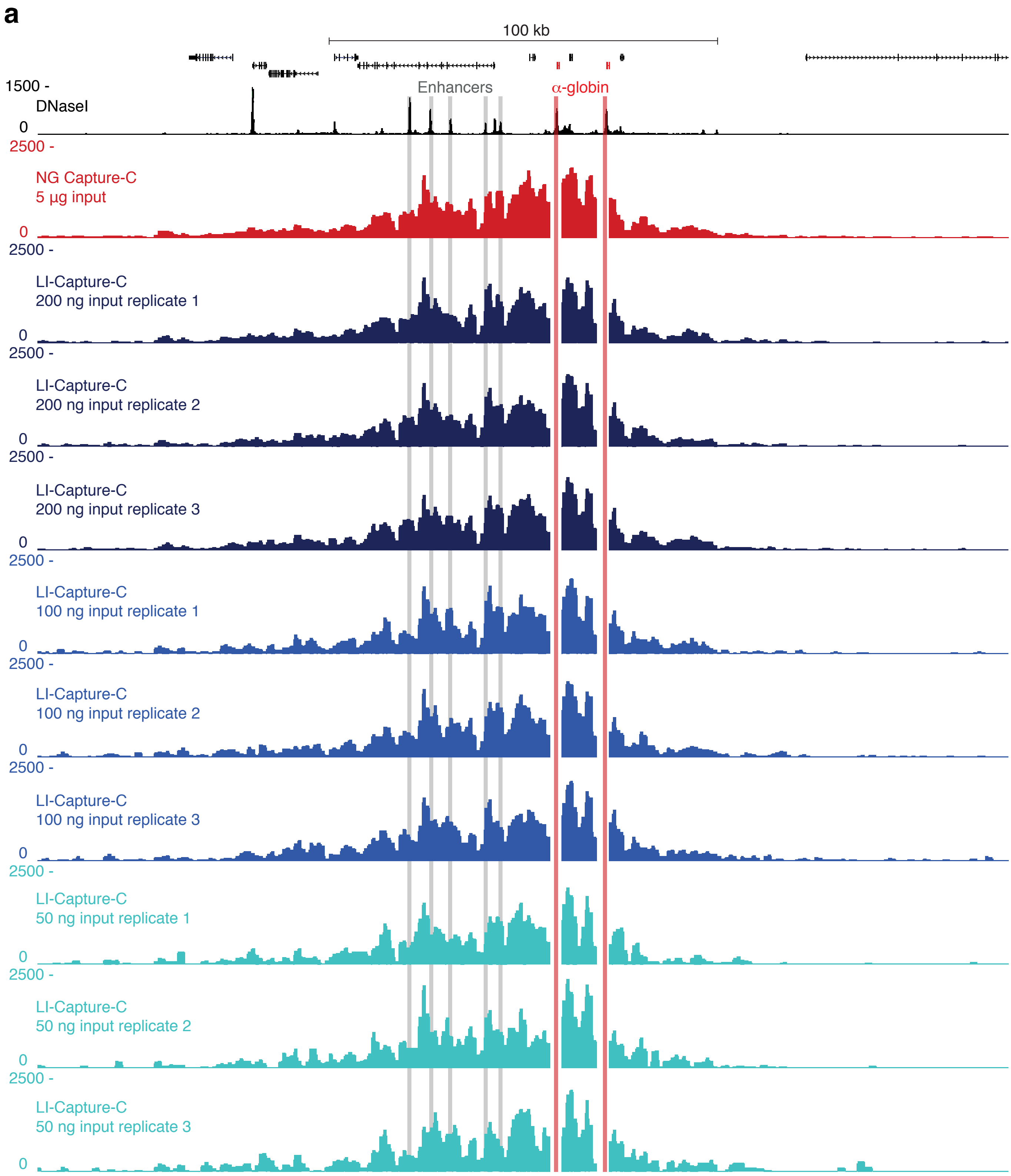

(Legend on next page.)

Supplementary Figure 7

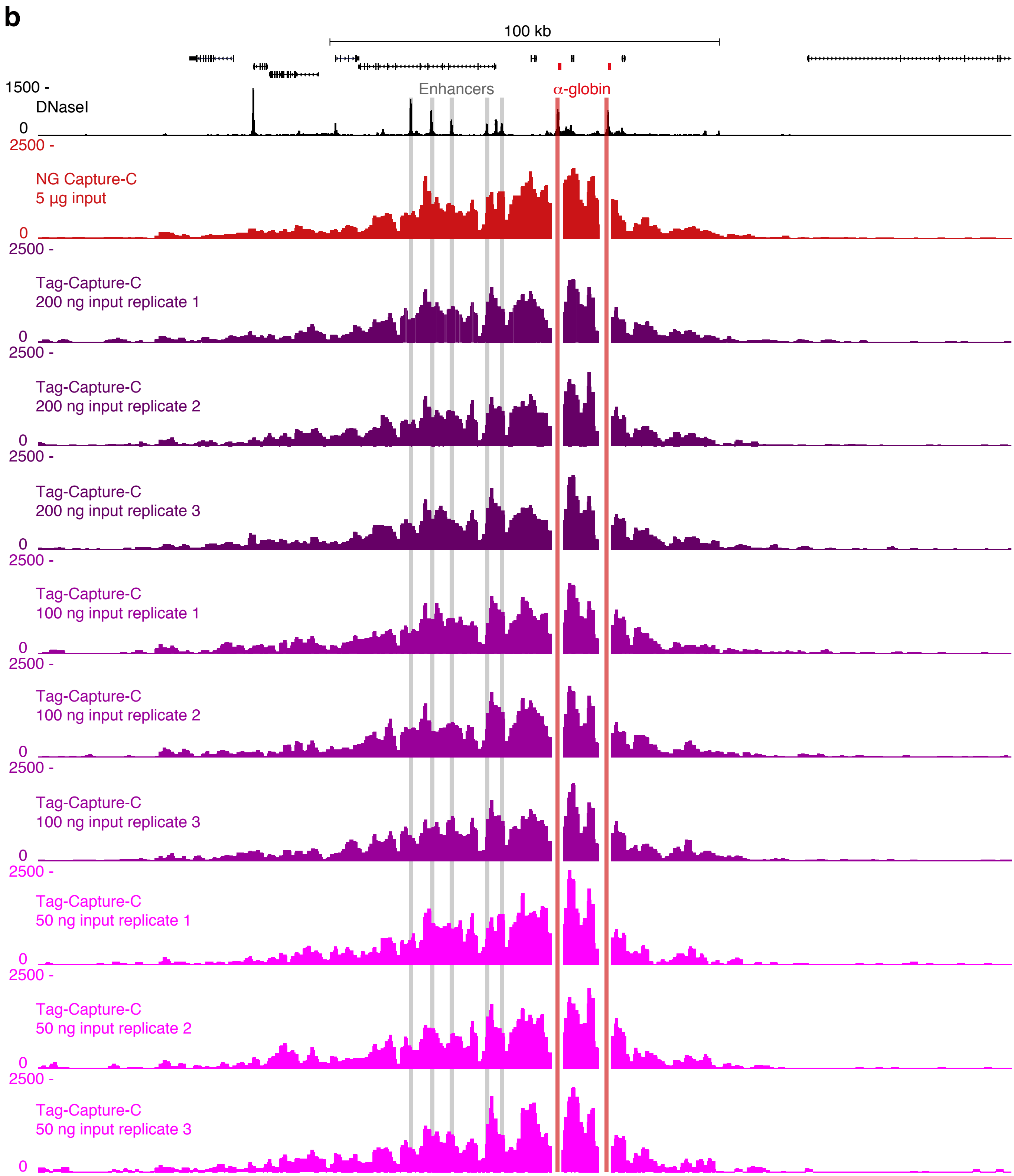

Supplementary Figure 7. Windowed interaction profiles of individual LI-Capture-C and Tag-Capture-C replicates.
Interaction profiles from the viewpoint of the α-globin promoters (highlighted in red) from individual replicates, generated from decreasing amounts of 3C libraries (prepared from primary erythroid cells) with **(a)** LI-Capture-C and **(b)** Tag-Capture-C. Profiles show normalized unique interactions with the α-globin promoters (highlighted in red) in a moving window (window size = 2 kb; window increment = 1 kb). The α-globin enhancers are highlighted in grey.

Supplementary Figure 8

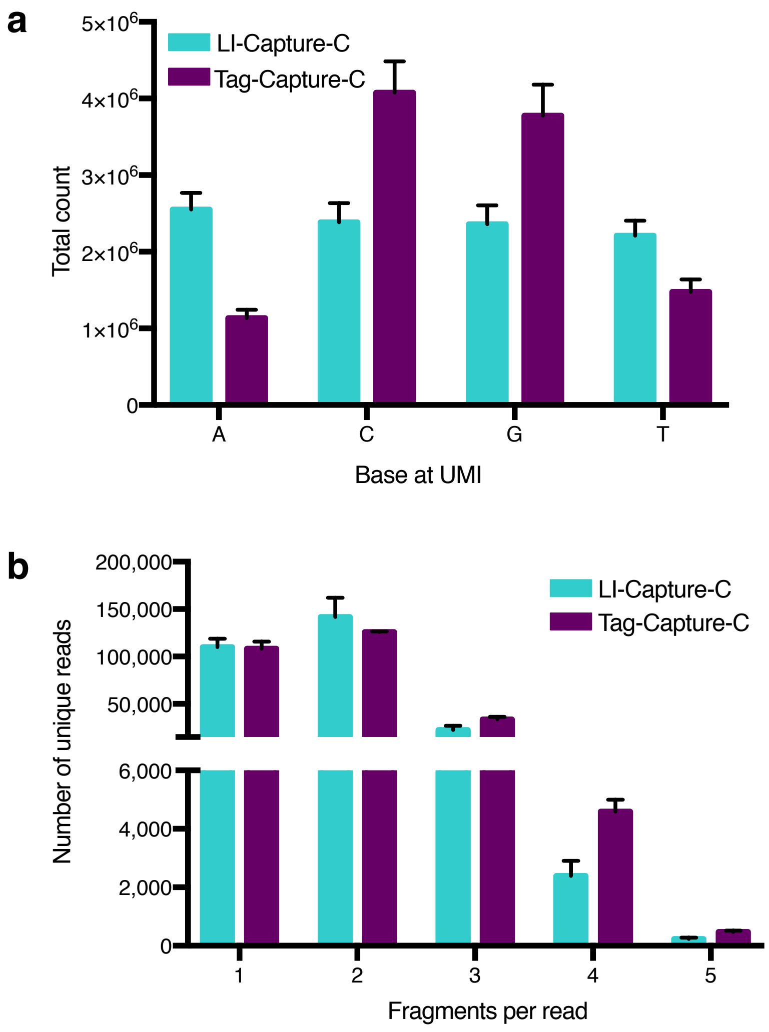

Supplementary Figure 8. Comparison of characteristics of LI-Capture-C and Tag-Capture-C libraries.
(a) Distribution of nucleobases at the sonication or tagmentation sites that are used as UMIs for PCR duplicate removal in LI-Capture-C and Tag-Capture-C, respectively. Bars represent average counts and standard errors of three replicate experiments using 200 ng 3C library.
(b) Comparison of the number of restriction fragments detected per unique read in LI-Capture-C and Tag-Capture-C experiments. Bars represent average read numbers and standard errors of three replicate experiments using 200 ng 3C library.

Supplementary Figure 9

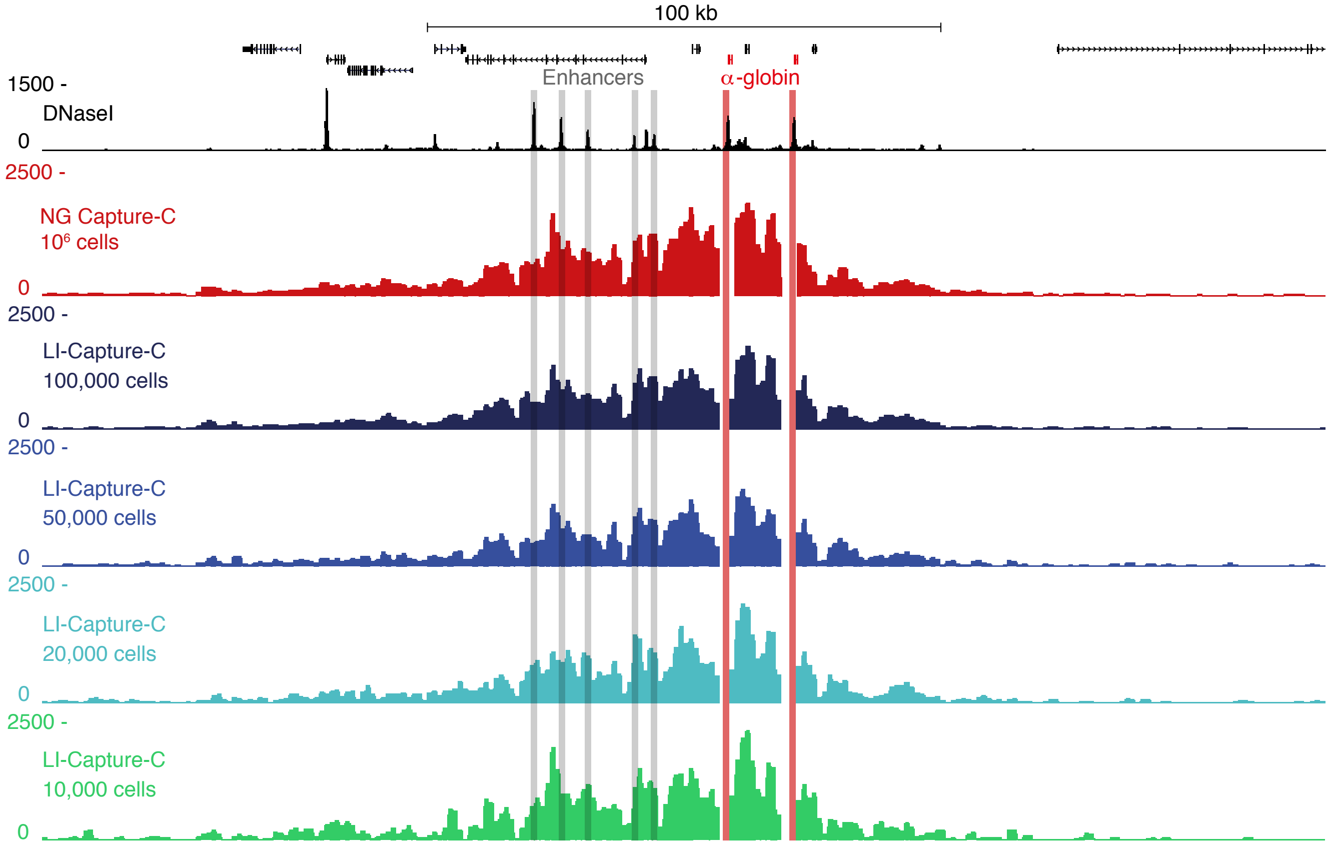

Supplementary Figure 9. Windowed interaction profiles generated with LI-Capture-C using 100,000, 50,000, 20,000 and 10,000 cells.
Windowed interaction profiles from the viewpoint of the α-globin promoters generated from decreasing numbers of primary erythroid cells with NG Capture-C (red) and LI Capture-C (blue / green). Profiles show normalized unique interactions with the α-globin promoters (highlighted in red) in a moving window (window size = 2 kb; window increment = 1 kb). The α-globin enhancers are highlighted in grey.

Supplementary Figure 10

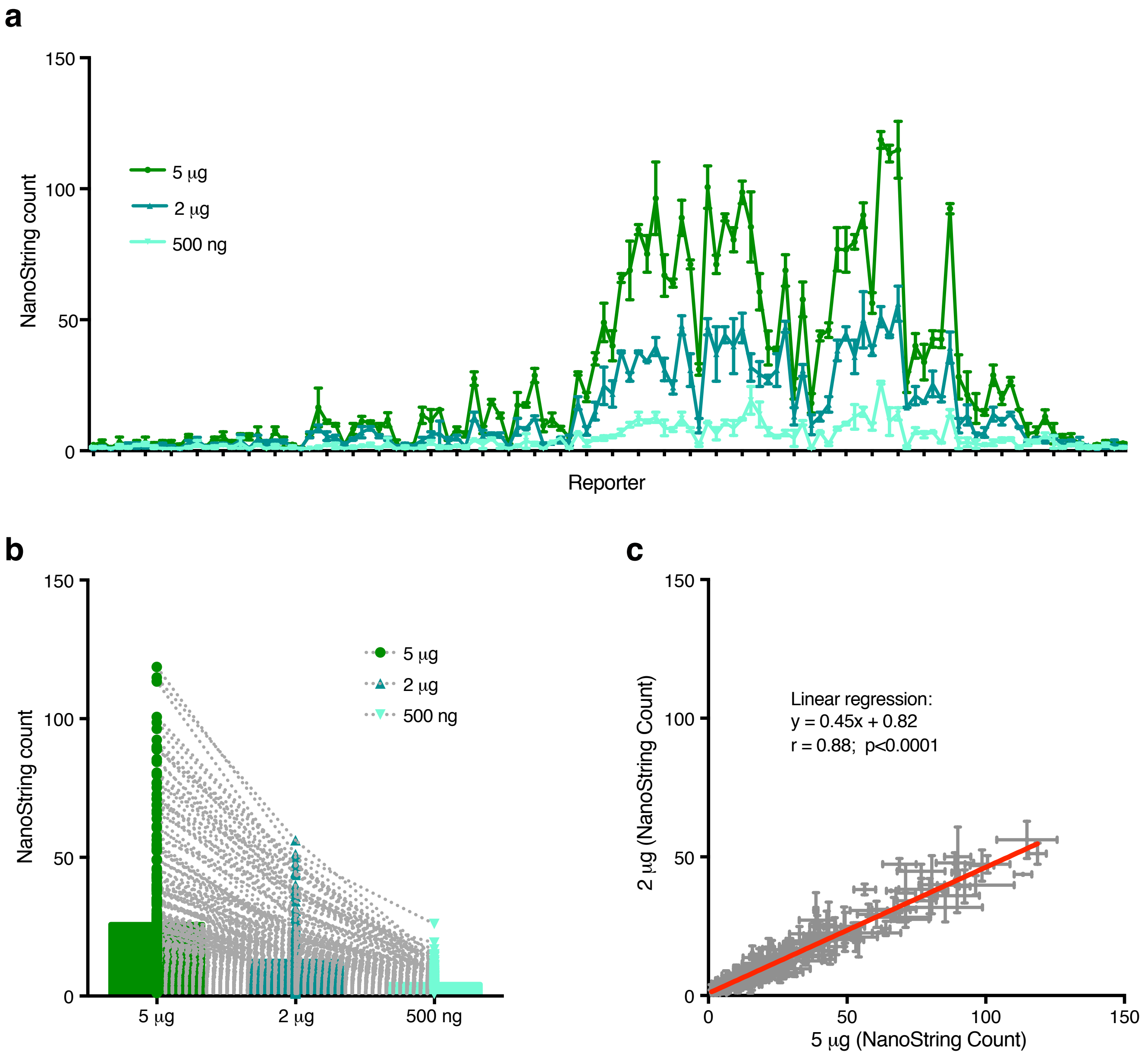

Supplementary Figure 10. Quantification of chromosomal interactions by C-String.
(a) Comparison of NanoString counts between C-String experiments using 5 µg (green), 2 µg (teal) and 500 ng (turquoise) 3C library (prepared from primary erythroid cells) input. The NanoString counts represent the average of two replicates, normalized for internal controls, and error bars indicate the standard deviation.
(b) Combined strip chart and bar graph showing the relationship between the NanoString count and the amount of 3C library input. The strip charts show individual data points from reporter probes in each sample. The grey dotted lines link data points from the same reporter probes. The bar graphs represent the mean counts.
(c) Scatter plot showing the correlation between the NanoString counts in C-String experiments using 5 µg or 2 µg 3C library input. The bars represent the range of the two replicates. The red line shows the linear regression line.

Supplementary Figure 11

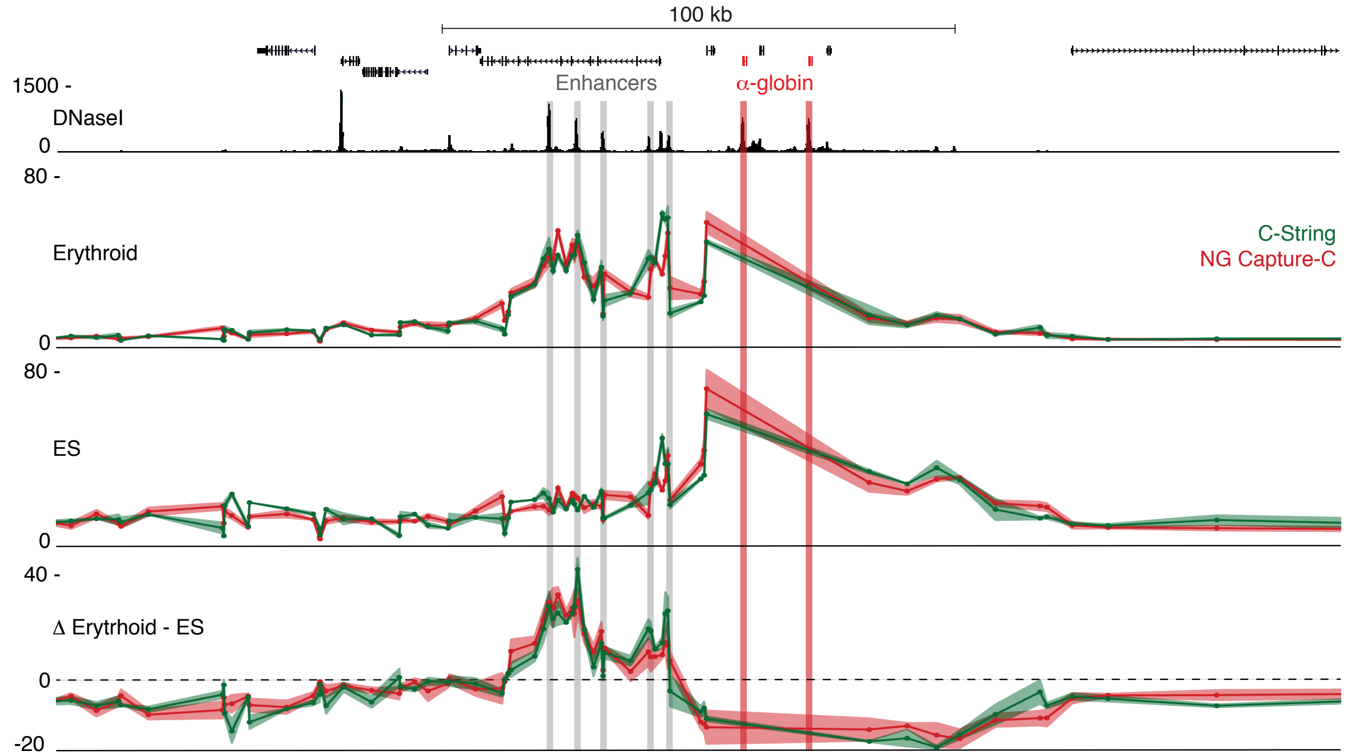

Supplementary Figure 11. Comparison of C-String and NG Capture-C interaction profiles of the α-globin locus in primary erythroid and ES cells.
Comparison of normalized interaction frequencies detected by C-String (5 µg input; green) and NG Capture-C (red) from the viewpoint of the α-globin promoters (highlighted in red). Interaction profiles in primary erythroid and ES cells are shown in the top and middle panel, respectively. The profile at the bottom shows a differential track, in which the profile in ES cells has been subtracted from the erythroid profile, to highlight tissue-specific interactions. The α-globin enhancers are highlighted in grey.

Supplementary Figure 12

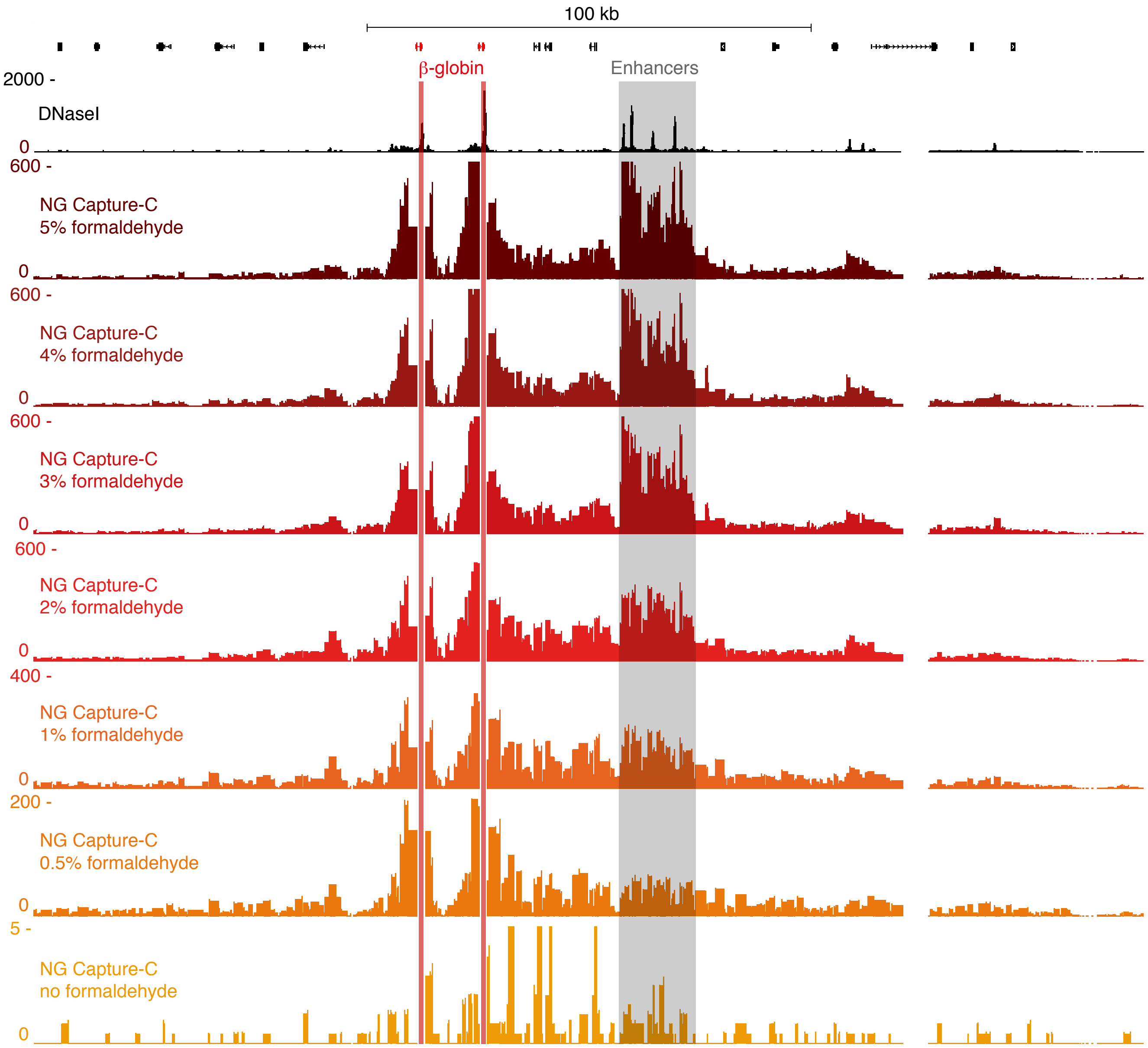

Supplementary Figure 12. β-globin NG-Capture-C profiles do not depend on the degree of formaldehyde fixation.
Comparison of NG Capture-C interaction profiles generated from the viewpoint of the β-globin promoters in primary erythroid cells that have been fixed with different concentrations of formaldehyde. Profiles show the mean number of unique interactions per restriction fragment from technical duplicates, normalized for a total of 100,000 interactions genome-wide, with the scales adjusted for the different conditions.

Supplementary Figure 13

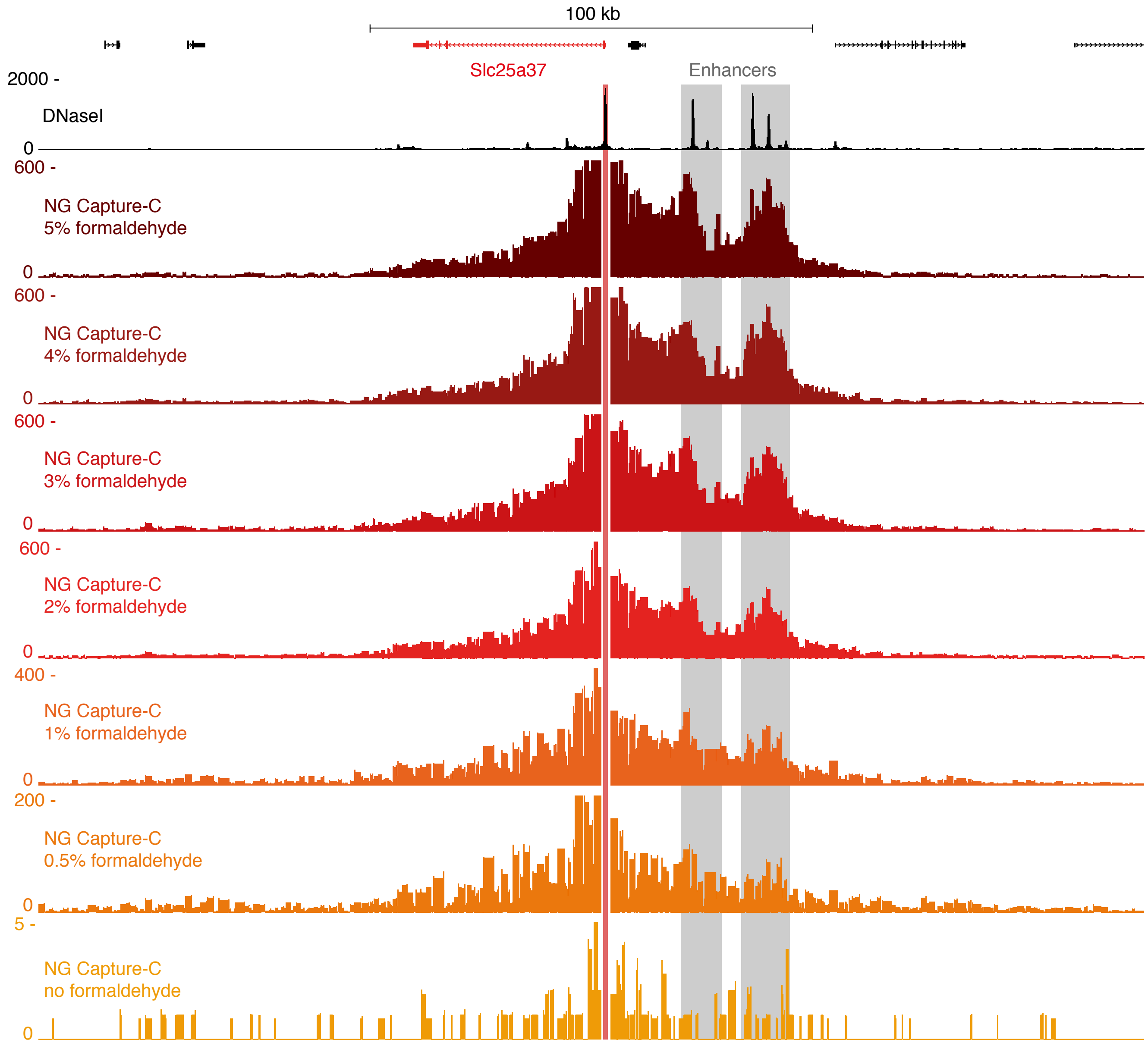

Supplementary Figure 13. Mitoferrin-1 NG-Capture-C profiles do not depend on the degree of formaldehyde fixation.

Comparison of NG Capture-C interaction profiles generated from the viewpoint of the Mitoferrin-1 promoter in primary erythroid cells that have been fixed with different concentrations of formaldehyde. Profiles show the mean number of unique interactions per restriction fragment from technical duplicates, normalized for a total of 100,000 interactions genome-wide, with the scales adjusted for the different conditions.
